# Coatings on Lithium Battery Separators: A Strategy to Inhibit Lithium Dendrites Growth

**DOI:** 10.3390/molecules28237788

**Published:** 2023-11-26

**Authors:** Huchao Cheng, Ruiqin Tan, Jia Li, Jinhua Huang, Weijie Song

**Affiliations:** 1 Faculty of Electrical Engineering and Computer Science, Ningbo University, Ningbo 315211, China; 2211100307@nbu.edu.cn; 2 Ningbo Institute of Materials Technology and Engineering, Chinese Academy of Sciences, Ningbo 315201, China; lijia@nimte.ac.cn (J.L.); huangjh@nimte.ac.cn (J.H.)

**Keywords:** lithium metal battery, lithium dendrites, separator modification, cycling reversibility

## Abstract

Lithium metal is considered a promising anode material for lithium secondary batteries by virtue of its ultra-high theoretical specific capacity, low redox potential, and low density, while the application of lithium is still challenging due to its high activity. Lithium metal easily reacts with the electrolyte during the cycling process, resulting in the continuous rupture and reconstruction of the formed SEI layer, which reduces the cycling reversibility. On the other hand, repeated lithium plating/stripping processes can lead to uncontrolled growth of lithium dendrites and a series of safety issues caused by short-circuiting of the battery. Currently, modification of the battery separator layer is a good strategy to inhibit lithium dendrite growth, which can improve the Coulombic efficiency in the cycle. This paper reviews the preparation, behavior, and mechanism of the modified coatings using metals, metal oxides, nitrides, and other materials on the separator to inhibit the formation of lithium dendrites and achieve better stable electrochemical cycles. Finally, further strategies to inhibit lithium dendrite growth are proposed.

## 1. Introduction

In recent years, with the global energy surge and environmental degradation, the development of green energy technologies has become imminent [1]. In particular, the rapid development of new energy-electric vehicles and portable electronic devices requires batteries with higher performance energy density and stable cycling [2,3,4]. Traditional lithium-ion batteries have been widely used because of their high energy density, long cycle life, high potential difference, and low self-discharging rate, especially since LiCoO_2_ was proposed as a new cathode material. LiMn_2_O_4_ and LiFePO_4_ cathode materials have also gradually been commercialized under the discovery of researchers [5,6,7,8,9]. Until now, the NCM series lithium-ion batteries have had a higher energy density [10]. However, the theoretical capacity of the graphite anode of commercial lithium-ion batteries is only 372 mAh g^−1^, which is far from being able to meet the demand for high-energy-density batteries [11]. The researchers explored the possibility of replacing the graphite anode with a silicon anode, but because the huge volume expansion and contraction of the silicon anode can cause the electrode to powder, they combined silicon with carbon to inhibit it [12,13,14]. Tin-based negative [15,16], sodium-ion batteries [17,18], zinc-ion batteries [19,20], and capacitive energy storage devices have also been proposed by researchers to replace lithium-ion batteries to relieve energy pressure [21,22]. With its extra-high capacity (3860 mAh g^−1^) and the lowest negative electrochemical potential (−3.040 V), Li metal is regarded as the “Holy Grail” electrode and receives extensive research attention. However, lithium metal batteries, including lithium–sulfur batteries [23,24], lithium–oxygen batteries [25,26], and lithium–selenium batteries [27,28], need to overcome many problems before industrialization. The SEI layer generated during the electrochemical cycling of the battery mainly prevents the reaction between the electrolyte and the electrode material. However, due to its high reactivity, lithium metal is extremely easy to react with the electrolyte, which leads to the continuous rupture and reconstruction of the SEI layer during cycling, increases the impedance of the battery, and reduces the cycling efficiency [29]. Conventional commercial lithium-ion batteries work by embedding and dragging lithium ions back and forth in the graphite anode during charging and discharging [30]. Lithium metal batteries work by plating and delighting lithium ions on the lithium metal anode, which is more likely to produce lithium dendrites. The generation of lithium dendrites provides a localized hot spot on which subsequent lithium ions will tend to be deposited, which is known as the tip effect [31]. The continuous growth of lithium dendrites reduces the electrical connection with the contact substrate, leading to an increase in the impedance and polarization of the battery cycle. Finally, the dendrites and the substrate undergo a stripping process, resulting in dead lithium and a decrease in the capacity of the battery. Therefore, in order to inhibit the growth of lithium dendrites, researchers have made great efforts and adopted lots of strategies, such as the construction of an artificial SEI layer [32,33,34], the introduction of solid electrolytes [35,36,37], electrolyte additives [38,39,40], the construction of a three-dimensional lithium metal anode skeleton [41,42,43], separator modification [44,45,46], and an artificial protective layer for the lithium metal anode [47,48,49]. In general, we focus on the practical application of materials and the feasibility of industrialization after material research is mature. Constructing a modified coating on the surface of the separator is an efficient way to inhibit the growth of dendrites, which can be achieved by magnetron sputtering, thermal evaporation, electroplating, sol–gel, and other methods. This paper reviews the preparation of coatings using various metals, oxide, nitride, and other materials on separators in recent years and summarizes their mechanisms.

## 2. Formation of Lithium Dendrite and Inhibition Principle

**Figure 1:**
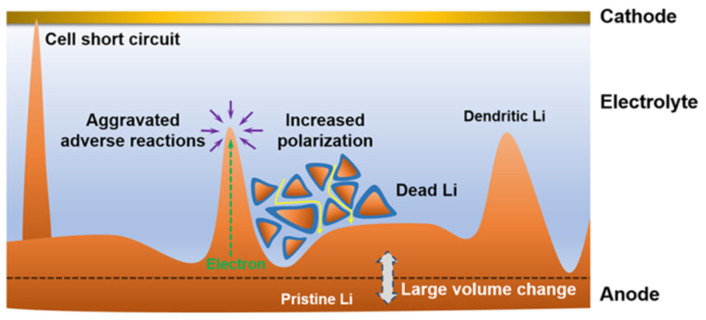
Scheme of a dilemma for Li metal anode [53].

Lithium metal batteries exhibit a higher propensity for dendrite growth compared to lithium-ion batteries due to the lithium-plating mechanism at the anode, in contrast to the intercalation principle employed in lithium-ion batteries [50,51]. Dendrites’ growth invariably originate from the negative electrode and extends towards the separator [52]. The battery separator mainly plays the role of isolating the anode and cathode and ensuring a certain ion transmission. The porosity distribution, pore size, electrolyte wettability, and mechanical properties of the separator will directly affect the battery’s performance. The porous structure of conventional commercial lithium battery separators (PP, PE), characterized by varying pore sizes, induces non-uniform lithium ion flux across the separator–anode interface, resulting in uneven electric field distribution, excessive electrolyte consumption, depletion of active lithium, and ultimately battery short circuit, particularly under conditions of high current density, high capacity, and elevated temperatures (Figure 1) [53].

Coatings of different materials (metals, oxides, nitrides, etc.) on the separator have good mechanical properties and can promote the uniform passage and deposition of Li^+^, which effectively inhibits the growth of lithium dendrites. The inhibition principles of lithium dendrites are as follows: (i) Enhancing the hardness of the separator; (ii) enhancing electrolyte wettability; (iii) regulating Li^+^ flux; and (iv) uniforming nucleation of Li^+^. We will discuss the application of these coatings to batteries.

## 3. Modification by Metal Coatings

**Figure 2:**
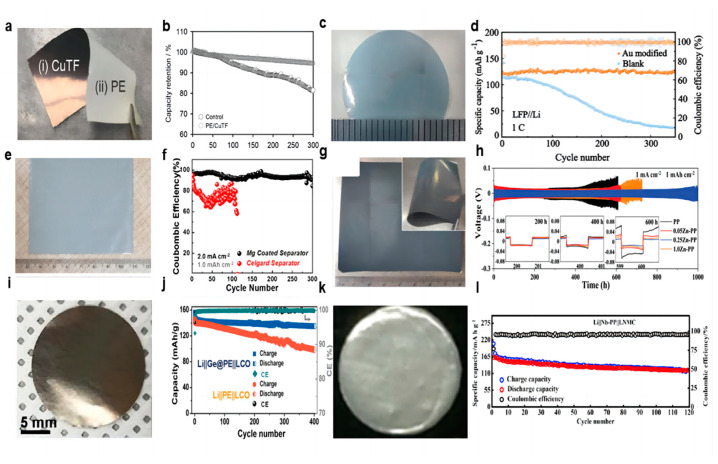
Optical photographs of coatings. (**a**) Cu [54], (**c**) Au [55], (**e**) Mg [56], (**g**) Zn [57], (**i**) Ge [58], (**k**) Nb [59]. (**b**) Capacity retention with cycle numbers at 1 C rate [54]. (**d**) Cycling stability of the Li||LFP cells with Au-modified and blank separators (the light colours represent specific capacity and the dark colours represent coulombic efficiency) [55]. (**f**) Comparison of CE of Li plating/stripping on Cu foil at a current density of 2.0 mA cm^−2^ fitted with a capacity of 1.0 mAh cm^−2^ [56]. (**h**) Cycling performance of symmetrical cells at a current density of 1.0 mA cm^−2^ fitted with a capacity of 1.0 mAh cm^−2^ [57]. (**j**) Cycling performance of the Li||Ge@PE||LCO and Li||PE||LCO batteries at a current density of 100 mA g^−1^ [58]. (**l**) Charge–discharge capacity and Coulombic efficiency of LNMC cathode with Nb-PP separator [59].

Copper metal is electrochemically inert and does not react easily with lithium, which is widely used as an anode collector material for lithium batteries to obtain better electron collection. The electronic insulation of the lithium battery separator itself leads to a more difficult charge transfer at high current densities. Lee et al. [54] deposited an ultra-thin copper film (70 nm) on the negative side of the separator using magnetron sputtering (Figure 2a), which does not penetrate to the other side of the separator and does not block the pores of the original separator. The copper coating acts as an upper current collector for a lithium metal, which reduces the local current density by increasing the surface area of lithium deposition, provides more electron transfer for dead lithium, and reduces the loss of battery capacity to a certain extent. A portion of Li^+^ will be deposited on top of the copper-modified separator and finally merge with the lithium growing from the negative electrode to grow along the surface of the negative electrode. In the Li-LCO full battery test, after 280 cycles, the battery with the Janus separator maintained 95% of its initial discharge capacity, while the control battery maintained only 83%. It is also noteworthy that the control battery lasted only 92 cycles when evaluated based on 95% capacity retention, suggesting that a simple CuTF coating on the PE separator can increase cycle life by a factor of approximately three (Figure 2b). The CuTF coating can also effectively prevent heat shrinkage. After heat treatment at 140 °C for 20 min, the dimensional shrinkage of the PE/CuTF separator is only ≈16%, which maintains the mechanical properties of the diaphragm even at high temperatures, while the dimensional shrinkage of the bare PE separator is as high as ≈47%.

As a precious metal, gold has good chemical stability, and gold nanoparticles have unique photoelectric and physical properties. Ma et al. [55] used density functional theory to calculate the adsorption energy of Li atoms on Au (−3.058 eV) and Cu (−2.410 eV) to study the chemical affinity of Au to Li and proved that Au nanoparticles can be used as nucleating seeds to promote Li deposition after Au nanoparticles have good lipophilic properties. Ma’s colleagues used DC magnetron sputtering to plate a thin film of gold nanoparticles on one side of the separator to control the growth direction of the lithium dendrites (Figure 2c). The uniformly deposited lithium has low Gibbs free energy. In the first electrochemical cycle, the formation of a lithium–gold alloy builds a uniform electric field between the separator and the negative electrode, which helps to form a uniform distribution of Li^+^, eliminating the concentration gradient of ions. In the Li||Cu cell test, at 0.5 mA cm^−2^ current density and 1 mAh cm^−2^ capacity, the blank separator nucleation overpotential was as high as 74.3 mV, while the Au-modified membrane nucleation overpotential was only 21.4 mV. When the lithium and LFP were assembled into a full battery, the battery using the modified separator showed an excellent capacity of 123.2 mAh g^−1^ and achieved a high capacity retention rate of 97.8% after 350 cycles at 1 C rate (Figure 2d). In addition, in the battery test of Li||NCM811, at a rate of 1 C, the modified separator battery maintained an initial capacity of 114.4 mAh g^−1^ and achieved a capacity retention of 75.1% after 300 cycles. Although gold nanoparticles show good lipophilicity and can provide abundant nucleation sites in the process of lithium deposition, the feasibility of industrialization is very low due to their high cost.

Because metal magnesium (Mg) has a huge solid solubility of Li and lithiophilic character, Liu et al. [56] used DC magnetron sputtering to plate a thin film of Mg metal nanoparticles on one side of the separator as an ideal nucleation site for lithium deposition (Figure 2e). The functional integrity and three-dimensional porous structure of the separator were preserved during sputtering. The nucleation overpotential of Mg is only 6 mV, far lower than that of bare copper (29 mV). The behavior of Li||Cu with a Mg-modified separator and a blank separator was compared under a current density of 2 mA cm^−2^ and a capacity of 1 mAh cm^−2^. After 300 cycles, the cells using the Mg-modified separator can still show a high CE of 93% (Figure 2f). LCO was selected as the cathode of the lithium metal battery for testing at the ratios of 1 C and 2 C. At a ratio of 1 C, the battery using a Mg-modified separator can provide a high capacity of 131 mAh g^−1^ on the first cycle and maintain 80% of the 104 mAh g^−1^ capacity after 400 cycles. For the blank separator, after 330 cycles, the battery had a capacity of 75.9 mAh g^−1^, only 60.2% of the initial capacity. At a high rate of 2 C, the battery using the Mg-modified separator maintained 70.6% capacity after 500 cycles, while the blank separator battery maintained only 20.2% capacity after 350 cycles.

Metal zinc (Zn), as a widely distributed element in nature, also has a lower cost in industrial use. The researchers found that the separator’s own uneven macropore characteristics lead to uneven Li^+^ flux through the electrolyte process, and the construction of the artificial SEI layer is difficult to solve. Lin et al. [57] proposed a Zn nanopartical film as the modified layer on the separator using DC magnetron sputtering (Figure 2g). In the initial electrochemical lithium plating process, lithium and Zn nanoparticles on the separator will form a Li–Zn alloy layer in situ. The Li–Zn alloy layer can be used as a rectifier layer for Li^+^ and a lithiophilic layer. The rectifier layer can provide a rapid diffusion path for Li^+^ in the electrolyte, homogenize the Li^+^ flux, and thus form a uniform electric field at the interface. The modified separator has a uniform nanopore, which enhances the wettability of the electrolyte. The alloy layer nucleates lithium uniformly on its surface due to its high amphiphilicity, causing lithium to grow in the reverse direction toward the anode, which changes the traditional direction of lithium dendrite growth from the negative electrode to the separator. In the Li||Li symmetric cell test, under the current density of 1 mA cm^−2^ and the capacity of 1 mAh cm^−2^, the 0.25 Zn-PP separator showed the most stable performance, and the cycle time is as long as 1000 h (Figure 2h). The modified separator was paired with a commercial LFP cathode and a lithium metal anode for a full-cell test. At a high cathode loading mass of 11 mg cm^−2^ at a magnification rate of 1 C, it showed a high reversible capacity of 135 mAh g^−1^ and a high capacity retention rate of 95.4%. Even when the cathode loading mass reaches the commercial value of 19.72 mg cm^−2^, a significant reversible capacity of 144 mAh g^−1^ can still be maintained after 120 cycles at a rate of 0.33 C.

Yue et al. [58] used a facile thermal evaporation technique to plate Germanium (Ge) film on the PE separator as the modified layer (Figure 2i). In the process of the Li–Ge reaction, the Ge interlayer spontaneously formed a folded dense SEI layer and firmly fixed it on the surface of the modified separator, which can provide a more active surface area for Li^+^ deposition to achieve superior electrochemical performance. In the test of the Li||Cu half cell, under the current density of 1 mA cm^−2^ and capacity of 1 mAh cm^−2^, CE was maintained at about 90% after 150 cycles using the modified separator, and the cycle of the cell using the blank separator stopped directly after 90 cycles. After 400 cycles, the capacity of the Li|| LCO full cell using the modified separator was maintained at 135 mAh g^−1^, with a retention rate of 92% under the current density of 100 mA g^−1^. However, the specific capacity of the control group was only 100 mAh g^−1^ (Figure 2j).

Din et al. [59] used RF magnetron sputtering to prepare a Nb film-modified separator (Figure 2k). The high mechanical strength of the Nb coating is conducive to supporting the structural integrity of lithium during electrochemical processes. The nano-metal Nb layer deposited on one side of the PP separator acts as an additional conductive agent to promote the electrochemical stripping/deposition of lithium. The Nb coating improves the wettability of the PP separator’s surface to the liquid electrolyte, enhances the Li^+^ flux, and improves the thermal stability of the membrane. The full cell using the modified separator had better charging–discharging cycle performance, and the initial discharge capacity at a rate of 0.2 C was 165 mAh g^−1^, and the reversible capacity after 120 cycles was 130 mAh g^−1^ (Figure 2l). The Nb coating not only enhances the transport of Li^+^, but it also plays a buffer role in the growth and penetration of Li dendrites.

There are several reasons why metal-coated modified separators can improve the cycling effect of lithium–metal batteries, including (1) providing additional conductive agents to increase electron transfer; (2) constructing a uniform electric field between the separator and the anode; (3) enhancing ionic rectification by an in situ lithiation-reactive alloy layer; (4) providing nucleation sites; (5) constructing an artificial SEI layer; and (6) enhancing the separator’s wettability to the electrolyte. Most of these modified separators can be prepared using magnetron sputtering, possessing the potential for large-scale preparation, and the nanoscale coatings do not additionally add weight to the separator to lose energy density. The information of separator-coating metal materials, fabrication methods, separators, electrolytes and full cell electrochemical results is listed in the Table 1.

## 4. Modification by Metal Oxides Coatings

**Figure 3:**
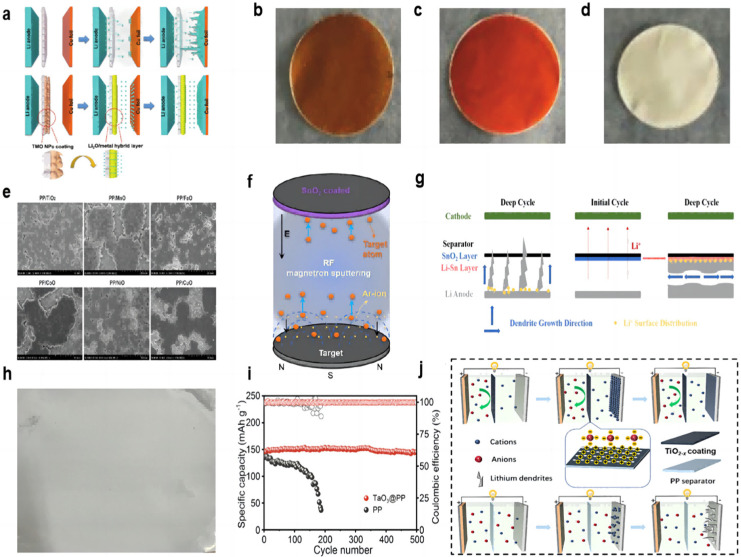
(**a**) Schematic view of the Li deposition behavior for cells without and with metal/Li_2_O coating [60]. Optical photographs of coatings. (**b**) Fe_3_O_4_ [60], (**c**) Fe_2_O_3_ [60], and (**d**) NiO [60]. (**e**) Top view SEM images of deposited Li on Cu foils after the first deposition in the Li||Cu cells at 0.5 mA cm^−2^ for 2.0 mAh cm^−2^ [61]. (**f**) Schematic diagram of the synthetic route for the SnO_2_-modified separator [62]. (**g**) Schematic of dendrite growth in cells with a blank separator and a SnO_2_-modified separator [62]. (**h**) Optical photographs of the TaO_3_ coating [63]. (**i**) Long-term cycling performance of full cells with PP separator and TaO_3_@PP separator at 0.5 C (the dark colours represent coulombic efficiency and the light colours represent specific capacity) [63]. (**j**) Schematic illustrations of the ionic transport regulation process on TiO_2−x_@PP and PP [64].

Many transition metal oxides (TMO) can be directly converted to a uniform metal/Li_2_O hybrid structure during the electrochemical primary lithiation process (Figure 3a). The metal/Li_2_O hybrid interlayer has a high ionic conductivity to allow lithium ions to be redistributed on the electrode surface and inhibit dendrite growth. Huang et al. [60] used the method of vacuum filtration to prepare the coating of Fe_2_O_3_ and Fe_3_O_4_ and coated it on the PP separator (Figure 3b,c). The resulting hybrid interlayers have abundant grain boundaries and can provide uniformly distributed ion transport channels. Under experimental conditions with a current density of 3 mA cm^−2^ and a capacity of 1 mAh cm^−2^, the symmetrical cell with a hybrid layer can cycle for more than 400 h, while the blank separator cell shorts out after 100 h. In the full-cell test, at a rate of 0.5 C, the initial capacity of the full cell using the separator of Fe_3_O_4_ was 159.3 mAh g^−1^, and the capacity remained as high as 150.9 mAh g^−1^ after 250 cycles, with a retention rate of 94.7%. In addition, the initial capacity of the full cell using the separator of Fe_2_O_3_ was 143.6 mAh g^−1^, and the capacity retention rate was 98.3% after 250 cycles. NiO nanoparticles can also be used as the modified layer of the separator to form a hybrid layer of Ni/Li_2_O (Figure 3d). Under the conditions of a 1 mA cm^−2^ current density and a 1 mAh cm^−2^ capacity symmetric cell test, the cell with the NiO-modified separator was stably cycled for more than 500 h, and the polarization was reduced compared to the cell using blank separator.

Yan et al. [61] also found that TMO particles (including MnO, NiO, CoO, FeO, etc.) slightly dissolved in organic electrolytes and formed a composite layer of metal and Li_2_O in the initial electrochemical reaction. The reduced metal nanoparticles formed an SEI passivation layer in situ and provided nucleation seeds for lithium deposition at the interface. A total of 2 mAh cm^−2^ of lithium was deposited at a current density of 0.5 mA cm^−2^ using a separator coated with excessive TMO particles, and all lithium anode wafers exhibited a dendrite-free double-layer lithium coating (Figure 3e). Cells with separators coated with MnO and NiO showed denser spherical lithium deposition, and MnO has a lower cost. In the Li||Li symmetric cell test, the voltage distribution of the battery with the MnO-modified separator was stabilized at around 20 mV for 5000 h at a current density of 2 mA cm^−2^.

The metal oxide-modified layer can also react with lithium in situ to generate a lithium metal alloy layer that has a better affinity for lithium than pure lithium foil and copper, which reduces the nucleation overpotential. Ma et al. [62] used RF magnetron sputtering to prepare a SnO_2_-modified layer on the separator (Figure 3f). The in situ generation of a Li–Sn alloy layer can change the direction of lithium dendrite growth due to its high lithiophilicity (Figure 3g). In the Li||Cu half-cell test, at current densities of 1 mA cm^−2^ and a capacity of 1 mAh cm^−2^, the cell using the modified separator was able to cycle stably for more than 250 cycles and maintain a CE value of more than 97%. In the Li||Li symmetrical cell test, at a high current density of 5 mA cm^−2^ and a capacity density of 1 mA h cm^−2^, the cell had a stable cycling process of 300 h. In the Li||LFP full cell test, the discharge capacity of the cell with the SnO_2_-modified separator is 113.7 mAh g^−1^ in the initial cycle, and then the discharge capacity increases gradually and can finally be maintained at 126.8 mAh g^−1^ after 300 cycles.

Two-dimensional (2D) layered materials are good candidates for modified coatings for lithium–metal battery separators by virtue of their excellent electronic and mechanical strengths, and the thickness of the coated two-dimensional nanosheets is only on the order of nanometers, which does not significantly cause an increase in the thickness and weight of the modified separator. Chen et al. [63] prepared a suspension of prepared tantalum oxide (TaO_3_) nanosheets by vacuum filtration onto a PP separator, which was then washed several times with ethanol and left to dry at room temperature (Figure 3h). The nano-network structure of the prepared modified separator, on the one hand, can provide an open channel with almost the same size as Li^+^. Li^+^ of the same size can pass through the separator, and Li^+^ of larger size is selectively excluded, successfully mediating the Li^+^ and homogenizing the electric field. On the other hand, the strong electrostatic interaction between the inherently negatively charged TaO_3_ nanosheets and the Li^+^ contributes to the homogeneous Li^+^ flux, and the electrostatic repulsion with the anions better avoids the concentration polarization of the anions. In the 0.5 mAcm^−2^ current density and 1 mAh cm^−2^ capacity of Li||Li symmetric cell test, the cell with modified separator has an ultra-long cycle life of more than 900 h, with a very low and stable voltage profile hysteresis and a voltage polarization of only 10 mV. The control group had a rapid widening of the voltage polarization after 400 h of cycling. The LFP cathode, modified/blank separator, and lithium anode were assembled into a full cell. The TaO_3_@PP separator was used to exhibit an initial capacity of 147 mAh g^−1^ at a multiplication rate of 0.5 C and maintained a capacity of 145 mAh g^−1^ after 500 cycles. In contrast, the cell using a blank separator had an initial capacity of 135 mAh g^−1^ and maintained only 40 mAh g^−1^ after 190 cycles (Figure 3i).

The free migration of anions formed by the decomposition of the liquid electrolyte during lithium–metal battery cycling poses a number of serious problems, including concentration polarization, Joule heating, and performance differences at high rates. Researchers are looking for functional materials that can regulate the diffusion of cations and anions. Semiconductor TiO_2_ is often used as a protective layer. TiO_2_ with oxygen vacancies has Ti atoms near the oxygen vacancies with a localized excess of electrons, and the localized electrons have a strong attraction to lithium nuclei, favoring the dissolution of lithium nuclei from lithium clusters into isolated individuals, facilitating the migration of cations, and leading to the possibility of depositing more lithium ions more evenly throughout the cycle. Oxygen vacancies with a positive charge have a strong affinity for anions and can better inhibit anion migration (Figure 3j). An et al. [64] prepared TiO_2_ nanoparticles by the hydrothermal method. Subsequently, TiO_2−x_ (titanium dioxide with oxygen vacancy) was obtained by high-temperature calcination. Finally, the composite separator can be obtained by a simple coating method. In the Li||Li symmetric cell test, at the demanding conditions of 8 mA cm^−2^ current density and 8 mAh cm^−2^ capacity, it could cycle steadily for 800 h. In the full battery test with LFP as the working electrode, the capacity retention rate of the battery with the TiO_2−x_-modified separator after 400 cycles reached 97.4% at the rate of 1 C. At the rate of 4 C, it could still maintain a long life of more than 350 cycles, and the capacity retention rate was 93.9%. At low magnification rates of 0.2 C and 0.5 C, the battery capacity retentions were also 89.6% and 87.35%, respectively. In contrast, the battery using a blank separator has only 60.1%, 44%, 79.85%, and 74.88% capacity retention rates, respectively, under the test conditions of high or low rate.

The reasons for the metal oxide coating to improve the cycling performance of lithium–metal batteries are (1) the generation of high ionic conductivity Li_2_O in the lithiation reaction; (2) the reduced metal in the lithiation reaction of the metal oxide as a nucleation site; (3) the generation of the rational alloy layer; and (4) the properties of the metal oxide coating material itself. The information of separator-coating metal oxide materials, fabrication methods, separators, electrolytes and full cell electrochemical results is listed in the Table 2.

## 5. Modification by Nitride Coatings

**Figure 4:**
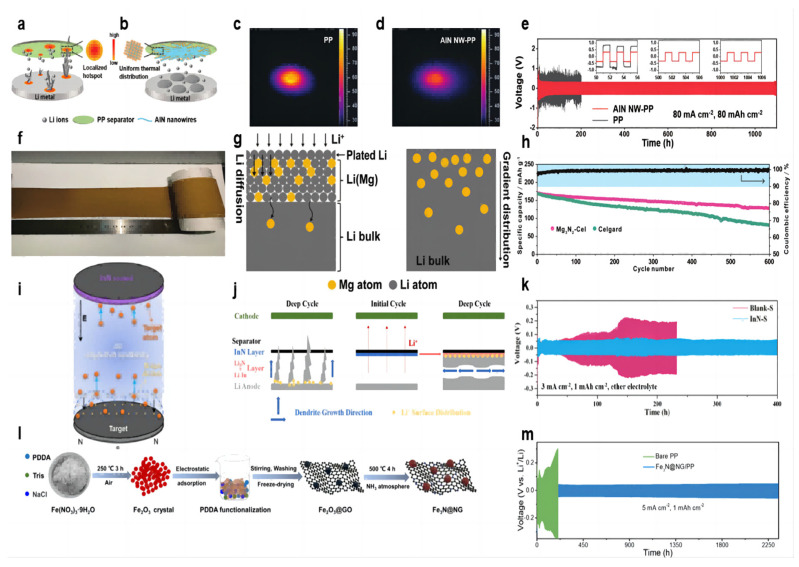
Schematic illustrations of Li deposition behavior. (**a**) With a blank PP separator [65] and (**b**) with an AlN NW-PP composite separator [65]. The temperature distributions for (**c**) the blank PP [65] and (**d**) AlN NW-PP [65]. (**e**) Electrochemical performances of symmetric Li||Li cells with AlN NW-PP or PP separator at 80 mA cm^−2^, 80 mAh cm^−2^ [65]. (**f**) Optical photograph of Mg_3_N_2_-Cel coating [66]. (**g**) Schematic illustrations of the Li diffusion inside the Li–Mg solid solution and the gradient distribution of Mg in the Li–Mg solid solution [66]. (**h**) Cycling performances of NCM622||Li cells at 0.5 C [66]. (**i**) Schematic separator of the InN thin film prepared by DC reactive magnetron sputtering [67]. (**j**) schematic of dendrite growth in cells with the blank separator and InN-modified separator [67]. (**k**) Cycle performance of Li||Li symmetric cells (ether electrolyte) assembled with Blank-S and InN-S at current densities of 3 mA cm^−2^ [67]. (**l**) Preparation of Fe_3_N@NG functional material [68]. (**m**) Voltage profiles for Li||Li symmetric cells with bare PP and Fe_3_N@NG/PP separators during galvanostatic cycles under 5 mA cm^−2^ with 1 mAh cm^−2^ [68].

The Joule heat generated during the cycling of lithium–metal batteries leads to uneven temperature distribution, and localized hot spots can make dendrite growth more uneven and severe, especially at high current densities and high multiplicities (Figure 4a). Guo et al. [65] prepared AlN nanowires by hot nitriding aluminum powder at 1200 °C and then coated the AlN nanowire slurry on the PP separator by convenient vacuum filtration to form the AlN NW-PP separator. The prepared separator has the following features: uniform temperature distribution and uniform lithium deposition; a porous structure for Li^+^ shuttling; and a high Young’s modulus to resist dendrite puncture (Figure 4b). The AlN coating has excellent thermal conductivity (319 W m^−1^ K^−1^) and high stiffness (23.7 GPa), and the morphology of nanowires builds a porous network. In the thermal conductivity tests, the temperature distributions were obtained by heating the AlN NW-PP or PP separator with an IR laser and recording the temperature distribution with an IR camera. There is a bright hotspot (≈90 °C) in the center of the PP separator with a large temperature gradient (Figure 4c). In contrast, the center temperature of the AlN NW-PP was drastically reduced to 60 °C, and the temperature distribution was quite uniform (Figure 4d). In the test of a Li||Li symmetric cell at 20 mA cm^−2^ current density and 3 mAh cm^−2^ capacity, the cell with an AlN NW-PP separator was able to cycle stably for more than 8000 h with voltage polarization stabilized at 78 mV. When the experimental conditions were changed to 50 mA cm^−2^ current density/25 mAh cm^−2^ capacity and 80 mA cm^−2^ current density/80 mAh cm^−2^ capacity, cells with an AlN NW-PP separator were able to cycle for more than 5000 h and 1000 h, respectively, which are well above the best performance of lithium–metal batteries to date (Figure 4e). In the long cycle test of the Li||LFP full cell, at a rate of 1 C, the battery with the AlN NW-PP separator was able to provide a specific capacity of 136 mAh g^−1^ with a capacity retention of 94.8% after 400 cycles, while the cell with a blank separator showed a specific capacity of 95.2 mAh g^−1^ after 400 cycles.

The lithium deposit layer in the lithium metal battery often plates on the surface of the lithium negative electrode because of the large current density and uniform ion flux, which makes it easier to generate lithium dendrites. So, the lithium diffusion into the lithium negative electrode can be a good solution to this problem. Among the traditional lithium alloy compounds, the Li–Mg alloy has outstanding advantages in lithium solubility, lithium diffusion kinetics, and lithium nucleation ability. Both lithium and magnesium can be alloyed at room temperature. Yan et al. [66] dipped the Mg_3_N_2_ slurry on one side of the commercial separator to form a uniform coating (Figure 4f). Batteries using a Mg_3_N_2_-Cel separator form a protective layer of a Li–Mg solid solution covering the lithium anode and Li_3_N during the initial electrochemical reaction (Figure 4g). The Li–Mg solid solution can diffuse subsequent Li^+^ into the lithium metal rather than the surface plating, avoiding the growth of lithium dendrites and parasitic reactions. At the same time, the diffusion coefficient of lithium increased with the increase in magnesium content in the Li–Mg solid solution. Li_3_N is a fast conductor of Li^+^, alleviating the concentration gradient of Li^+^. In the long-cycle Li–NCM622 full battery test, the initial capacity of the battery using the Mg_3_N_2_-Cel separator is 170 mAh g^−1^ at a rate of 0.5 C, and the reversible capacity after 600 cycles is 129 mAh g^−1^, with a capacity retention rate of 75.9%. In contrast, the battery using the blank separator dropped from 168 mAh g^−1^ to 81 mAh g^−1^ after 600 cycles, and the capacity retention rate was only 48.2% (Figure 4h).

The use of oxide coatings in modified separator batteries (such as Sn_2_O) will form a mixed modified layer of lithium–metal alloy and Li_2_O in situ with the lithium anode during the electrochemical cycle. Li_2_O can act as a good conductor of ion transfer, and Li_3_N has a higher ionic conductivity than lithium oxide. Ma et al. [67] modified the commercial separator with an InN coating using DC magnetron reactive sputtering (Figure 4i). The Li_3_N coating formed in situ a Li–In alloy and a Li_3_N binary-modified layer with the lithium anode (Figure 4j). The Li–In alloy provides a site for the formation of lithium dendrites on one side of the separator, inducing lateral growth of dendrites, and Li_3_N has higher ionic conductivity (10^−3^ S cm^−1^) (Figure 4h). In the Li||Cu cell test with 1 mA cm^−2^ current density and 1 mAh cm^−2^ capacity, the CE with modified separator cell stabilized at 97% after 200 cycles, and that with blank separator showed a downward trend after 70 cycles. In the Li||Li symmetric cell test, under the experimental conditions of 1 mA cm^−2^ current density, 1 mAh cm^−2^ capacity, and ether electrolyte, the cell using the modified separator can achieve a stable cycle of 600 h, and the overpotential is stable at about 50 mV. In contrast, the cell using the blank separator experienced a short circuit after 320 h. When the current density increased to 3 mA cm^−2^, the cell using the modified separator could achieve a stable cycle of 400 h, and the cell using the blank separator had a potential fluctuation after 50 h (Figure 4k). When the experimental conditions were converted to 1 mA cm^−2^/3 mA cm^−2^ current density and 1 mAh cm^−2^ capacity of ester electrolyte, the cell using the modified separator could be stably cycled for 550 h/180 h. In the full battery test, the InN-coated separator was able to increase its capacity retention rate to 92.1% after 300 cycles.

Transition metal oxide nanoparticle coatings have been proven to have a good effect on inhibiting lithium dendrites. As an emerging material, transition metal nitrides have a good effect on inhibiting the growth of lithium dendrites. Zhang et al. [68] explored the feasibility of transition metal nitrides as emerging materials to inhibit lithium dendrites. A shield protective coating was constructed by N-doped rGO-wrapped Fe_3_N nanoparticles (Figure 4l). The N-doped rGO matrix, with its robust mechanical strength and abundant heteroatomic nitrogen, effectively inhibited Li dendrite growth. Additionally, the Fe_3_N nanoparticles at the core of the structure exhibited high chemical stability, rapid ionic diffusion, low conversion reaction potentials, and superior electrochemical activity. These properties provide ample deposition sites for uniform Li deposition, reduce the local current density applied to the anode electrode, and promote the formation of a stable solid electrolyte film. A symmetric cell employing a Fe_3_N@NG-coated separator exhibited remarkable stability, operating for over 2300 h at current densities of 5 mA/cm^2^ with an overpotential of merely 55.8 mV (Figure 4m). Li||LFP full battery with Fe_3_N@NG-coated separator showed a higher capacity and an ultra-low capacity decay rate of 0.08% at 2 C rate compared to battery with bare separator.

In general, nitride-coated modified separators have excellent thermal resistance, thermal conductivity, and high mechanical hardness. They are able to resist dendrite puncture and achieve uniform lithium deposition through uniform heat distribution. TiN and SiN-coated modified separators have been reported for applications in lithium-ion batteries [69,70]. In addition, the in situ formation of lithium nitride/alloy hybrid layers is also favorable for uniform lithium deposition. The information of separator-coating nitride materials, fabrication methods, separators, electrolytes and full cell electrochemical results is summarized in the Table 3.

## 6. Modification by Other Coatings

For other material coatings in lithium metal battery applications, attention can be paid to these aspects: (1) the lithiation reaction of the material; (2) the properties of the material itself; (3) the enhancement of the material for the separator electron transfer capability; (4) the enhancement of the material for the SEI layer, and so on. The information of separator-coating other materials, fabrication methods, separators, electrolytes and full cell electrochemical results is summarized in the Table 4.

**Figure 5:**
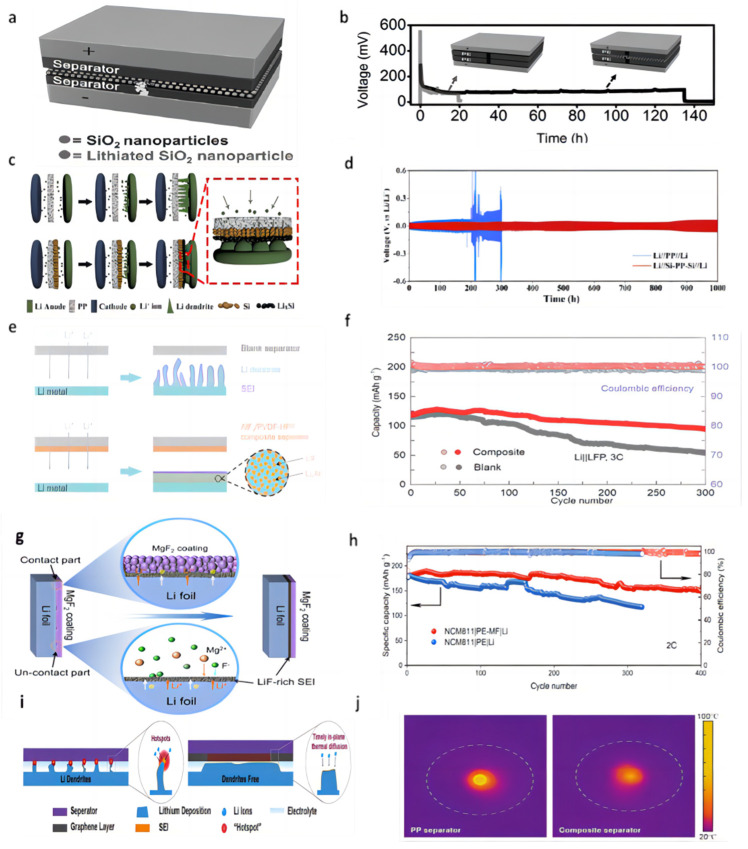
(**a**) A layer of silica nanoparticles was sandwiched by two layers of commercial polymer separators [71]. (**b**) Typical voltage versus time profile of a Li/Li battery with a conventional separator (grey curve) and the silica nanoparticle sandwiched trilayer separator [71]. (**c**) Schematic of the fabrication process of the Si-PP separator [72]. (**d**) Electrochemical performance of Li||Li symmetric cells at a current density of 0.5 mA cm^−2^ [72]. (**e**) Schematic of Li deposition with blank separator and AlF_3_/PVDF-HFP composite separator, LiF-rich SEI layer is coupled with Li−Al alloy to stabilize Li/electrolyte interface [73]. (**f**) Cell performance of the Li||LFP cells at the cycle ratio of 3 C [73]. (**g**) Schematic illustration of the formation of a continuous LiF-rich artificial SEI layer on the Li metal anode [74]. (**h**) Long-term cycling performance of NMC811||Li full cells with different separators at 2 C [74]. (**i**) Schematic of Li-ion deposition under localized-temperature hotspots and eliminated hotspots with reinforced thermal diffusion [75]. (**j**) The corresponding temperature distribution detection with a pristine separator and composite separator [75].

Cui et al. [71] successfully prepared a triple polyolefin separator stacked with coatings of silica nanoparticles obtained by the sol–gel method on both sides (Figure 5a). In the symmetric battery test, the battery was able to cycle for 135 h at a current density of 1 mA cm^−2^ using this three-layer sandwich structure separator (Figure 5b). The porous structure of the silica coating is conducive to the transportation of Li^+^ and the lithiation reaction (when dendrites grow into contact with silica nanoparticles, the silica nanoparticles react with lithium) and can prevent short-circuiting when dendrites penetrate the separator.

Chen et al. [72] constructed a “dendrite-eating” separator by coating a commercial PP separator with a slurry mixture consisting of 80 wt% Si particles and 20 wt% polyacrylic acid through a blade casting method (Figure 5c). In lithium–metal battery use, the silicon coating can react with lithium dendrites in a lithiation reaction to prevent short-circuiting the battery. The lithiation reaction also forms a silicon-rich SEI layer on the lithium surface, which serves as a lithium storage layer to replenish the lithium lost during cycling. In the Li||Cu cell test, the separator modified with Si coating was able to maintain a CE of 97.6% after 100 cycles under the experimental conditions of 0.5 mA cm^−2^ current density and 1 mAh cm^−2^ capacity, while the CE of the control group rapidly decreased to below 80% after 40 cycles. In a 0.5 mA cm^−2^ current density Li||Li symmetric cell, the voltage of the cell using a blank separator suddenly increased after 200 h. In contrast, the use of a Si-coated separator showed good cycling stability, with an average voltage hysteresis of less than 78.9 mV over 1000 h (Figure 5d). In full-cell testing with the LFP, the initial discharge capacity of the battery using a blank separator was 2.39 mAh cm^−2^ at a current density of 0.5 mA cm^−2^; after 65 cycles, the capacity rapidly dropped to 1.71 mAh cm^−2^. In contrast, the cell using a Si-coated separator was able to maintain a high capacity of 2.30 mAh cm^−2^ after 100 cycles.

Wang et al. [73] coated PVDF-HFP and aluminum fluoride particles uniformly onto one side of the Celgard separator. AlF_3_ can react with the highly active Li metal to form a lithium fluoride (LiF) coating in situ on the lithium metal surface, which helps to enhance the mechanical and electrochemical stability of the SEI layer, as well as having a high ionic conductivity that regulates lithium-ion fluxes along with the Li–Al alloy that is formed in situ (Figure 5e). In a 1 mA cm^−2^ current density and 1 mAh cm^−2^ capacity Li||Cu cell test, the CE of the cell with a blank separator began to decline after 20 cycles, and the cell with an AlF_3_-coated modified separator maintained a high CE of 98% after 120 cycles. In the Li||Li symmetric cell test, at a current density of 3 mA cm^−2^ and a capacity of 1 mAh cm^−2^, the polarization voltage of a cell with a blank separator increased rapidly by more than 400 mV, and the overpotential of a cell with an AlF_3_-coated modified separator was only 45 mV after 600 h of cycling. When the capacity was changed to 3 mAh cm^−2^, the cell with an AlF_3_-coated modified separator could be cycled stably for 400 h without any significant sudden change in voltage. Even at a high current density of 5 mA cm^−2^, the cell can be stably cycled for more than 200 h. In the full-cell test with LFP as the cathode and at the rate of 3 C, the capacity of the cell with a blank separator continued to decay after 100 cycles and dropped to 54 mAh g^−1^ after 300 cycles. In contrast to the cell with an AlF_3_-coated modified separator, the capacity retention was up to 78.3% after 300 cycles while maintaining a higher CE value (Figure 5f).

Tan et al. [74] coated the surface of the PE separator with MgF_2_ as a modified layer (PE-MF separator). When the MgF_2_ coating contacted the lithium anode directly, the SEI layer was formed in situ, which was composed of LiF and Mg (Figure 5g). Mg^2+^ has a higher standard reduction potential and serves as the nucleation site for bare Li in the early plating process. MgF_2_ has low solubility in the liquid electrolyte and can react with lithium metal that is not in contact with the MgF_2_ coating to form a continuous SEI layer. This layer can repair the small cracks in the SEI layer that occur during the charge and discharge processes. Under the symmetrical battery test with 1 mA cm^−2^ current density and 1 mAh cm^−2^ capacity, the cell with PE-MF separator could be used for stable cycling for more than 600 h, while the voltage hysteresis increased sharply after 250 h in the control group. The NCM811|PE-MF|Li full cell was able to maintain a capacity of 84.5% after 400 cycles at a rate of 2 C in comparison with the lower capacity retention of 66.4% after 320 cycles for the control group (Figure 5h).

Han et al. [75] uncovered that the heat production rate of the lithium metal tip region escalates with deposition time and overpotential. This instigates the occurrence of accumulated overpotential heat and localized temperature “hotspots” due to inadequate local thermal diffusion, which aggravates the undesirable irregular Li deposition and dendrite formation. A thermally conductive graphene-coated separator was constructed to eliminate these local hot spots (Figure 5i). The laminated 2D graphene sheets exhibited exceptional thermal diffusion properties. In a temperature distribution experiment using a laser beam as the heat source, the peak temperature of the PP separator reached 78 °C. Conversely, the maximum temperature of a graphene-coated separator was only 53 °C, and its heat dissipation encompassed a substantially larger area (Figure 5j). In the Li||Cu cell test, at a current density of 1 mA cm^−2^ and an areal capacity of 1 mAh cm^−2^, the CE of cells using the PP separator sharply drops to ≈70% after 120 cycles. When the current density was increased to 2 and 3 mA cm^−2^, the CEs of cells with the PP separator decreased to 76% after 80 cycles and 62% after only 60 cycles, respectively. The CEs of cells using a graphene-coated separator maintained >95% under the same cycling conditions and can maintain a CE of nearly 100% for 50 cycles even at a current density of 5 mA cm^−2^, a capacity of 5 mAh cm^−2^, and a temperature of 60 °C.

## 7. Conclusions and Outlook

In recent years, a variety of material coatings have been widely used in the modification of lithium metal battery separators. This paper reviews the application of various metals, oxides, nitrides, and other materials as separator coatings and the application principles, including enhancing the mechanical properties of the separator, constructing the uniform electric field between the separator and the anode, constructing the nucleation site, adjusting the ion flux, and controlling the anion concentration. There are still some problems to be solved with separator modifications.

(1) The different coating thicknesses will affect the energy density of the battery, as reported. This phenomenon needs further study, and the mechanism needs to be explored.
(2) Currently, partially modified coatings can be used as nucleation sites or lithiophilic sites to homogenize lithium deposition. If these nucleation sites and lithiophilic sites are covered by lithium deposition, does that mean that the modified coating loses its effect? Is there any way to improve this? More attention should be paid to the effective time constancy of the modified layer.
(3) At present, flexible wearable electronic devices are developing rapidly, and lithium metal batteries can be an ideal energy supply choice. So, flexible separator modifications and coatings need to be developed and investigated.

In this review, the different principles of inhibiting lithium dendrites by different materials have been comprehensively discussed. What we pursue is the practical applicability of the material after its feasibility is verified. The modification of single-layer coating has been able to have a certain effect, so the use of different materials to prepare double- or multi-modified layers to obtain better results is worthy of attention and consistent research.

## Figures and Tables

**Table 1:** Summary of references on metal coatings.

Material	Method	Separator	Electrolyte	Cathode	Loading Amount	Performance
Cu [54]	DC magnetron sputtering	PE separator (ND420)	1 M LiPF_6_ in EC/ DEC (1:1 *v*/*v*) with 1 wt% VC and 10 wt% FEC additives	LCO	0.5 mAh cm^−2^	1 C 280 cycles 95%
Au [55]	DC magnetron sputtering	Celgard 2325	Ether electrolyte: 1 M LiTFSI in DOL/DME (1:1 *v*/*v*) Ester electrolyte: 1.0 M LiPF_6_ in EC/DEC/EMC (1:1:1 *v*/*v*/*v*)	LFP NCM	2.4 mg cm^−2^ 2.8 mg cm^−2^	1 C 350 cycles 97.8% 1 C 300 cycles 75.1%
Mg [56]	DC magnetron sputtering	Celgard 2325	1 M LiPF_6_ in EC/DEC (1:1 *v*/*v*)	LCO	8.0 mg cm^−2^	1 C 400 cycles 80% 2 C 500 cycles 70.6%
Zn [57]	DC magnetron sputtering	Celgard 2500	1 M LiTFSI in DOL/DME (1:1 *v*/*v*) with 2 wt% of LiNO_3_ additive	LFP	4.0 mg cm^−2^ 11 mg cm^−2^ 19.2 mg cm^−2^	5 C 300 cycles 121 mAhg^−1^ 1 C 200 cycles 135 mAh g^−1^ 0.33 C 120 cycles 144 mAh g^−1^
Ge [58]	Thermal evaporation	PE separator	1.3 M LiPF_6_ in EC/DEC (1:1 *v*/*v*) with 5 wt% FEC additive	LCO	3 mg cm^−2^	100 mA g^−1^ 400 cycles 92%
Nb [59]	RF magnetron sputtering	Celgard 2325	1 M LiTFSI in DME/DOL (1:1 *v*/*v*)	LNMC	2.5 mg cm^−2^	0.2 C 120 cycles 130 mAh g^−1^

**Table 2:** Summary of references on metal oxide coatings.

Material	Method	Separator	Electrolyte	Cathode	Loading Amount	Performance
Fe_2_O_3_/Fe_3_O_4_ [60]	vacuum filtration	PP separator	1 M LiTFSI in DOL/DME (1:1 *v*/*v*) with 1 wt% LiNO_3_ additive	LFP	1 mg cm^–2^	0.5 C 250 cycles 94.7% (Fe_3_O_4_) 0.5 C 250 cycles 98.3% (Fe_2_O_3_)
MnO [61]	coating	PP separator	1 M LiTFSI in DOL/DME (1:1 *v*/*v*) with 1 wt% LiNO_3_ additive	LFP	/	1 C 600 cycles (with LiNO_3_ additive)
SnO_2_ [62]	RF magnetron sputtering	commercial Celgard separator	1 M LiTFSI and 0.2 M LiNO_3_ in DOL/DME (1:1 *v*/*v*) (Li||Li cell) 1 M LiPF6 in EC/DMC/DEC (1:1:1 *v*/*v*/*v*) (full cell)	LFP	/	1 C 300 cycles 126.8 mAh g^−1^
TaO_3_ [63]	vacuum filtration	commercial PP separator	1 M LiTFSI in DOL /DME (1:1 *v*/*v*) with 1 wt% LiNO_3_ additive	LFP	12 mg cm^−2^	0.5 C 500 cycles 145 mAh g^−1^
TiO_2−x_ [64]	coating	PP separator	1 M LiTFSI in DOL/DME (1:1 *v*/*v*) with 5 wt% LiNO_3_ additive	LFP	2.4 mg cm^−2^ (1 C/4 C) 9.24 mg cm^−2^ (0.5 C)	1 C 400 cycles 97.4% 4 C 350 cycles 93.9% 0.5 C 900 cycles 113.8 mAh g^−1^

**Table 3:** Summary of references on nitride coatings.

Material	Method	Separator	Electrolyte	Cathode	Loading Amount	Performance
AlN [65]	vacuum filtration	Celgard 2400	1 M LiTFSI in DOL/DME (1:1 *v*/*v*) with 2 wt% LiNO_3_ additive 1 M LiPF_6_ in EC/DMC (1:1 *v*/*v*)	LFP	2.0 mg cm^−2^	1 C 400 cycles 94.8%
Mg_3_N_2_ [66]	coating	Celgard separator	1 M LiPF_6_ in EC/DMC/DEC (1:1:1 *v*/*v*/*v*)	NCM622	3.0 mg cm^−2^	0.5 C 600 cycles 75.9%
InN [67]	DC magnetron sputtering	Celgard separator	1 M LiTFSI in DOL/DME (1:1 *v*/*v*) with 0.2 M LiNO_3_ additive 1 M LiPF_6_ in EC/DEC/DMC (1:1:1 *v*/*v*/*v*) with 5% FEC additive	LFP	/	1 C 300 cycles 92.1%
Fe_3_N@NG [68]	electro- static adsorption ammonization process	Celgard 2500	1 M LiTFSI in DOL/DME(1:1 *v*/*v*) with 1.0 wt% LiNO_3_ additive (Li||Cu,Li||Li) 1 M LiPF_6_ in EC/DEC (1:1 *v*/*v*) (full cell)	LFP	/	2 C decay rate of 0.08%

**Table 4:** Summary of references on other material coatings.

Material	Method	Separator	Electrolyte	Cathode	Loading Amount	Performance
SiO_2_ [71]	sol–gel method	PE separator	1 M LiPF_6_ in EC/DEC (1:1 *v*/*v*)	/	/	/
Si [72]	coating	Celgard 2325	1 M LiPF_6_ in EC and DEC (1:1 wt/wt) with 10 wt% FEC and 1 wt% VC additive	LFP	20.0 mg cm^−2^	0.2/0.5 mA cm^−2^ 100 cycles 2.30 mAh cm^−2^
AlF_3_ [73]	phase inversion method	Celgard separator	1.0 M LiTFSI in DOL/DME (1:1 *v*/*v*) with 0.2 M LiNO_3_ additive 1.0 M LiPF_6_ in EC/DC/DEC (1:1:1 *v*/*v*/*v*) (full cell)	LFP	/	3 C 300 cycles 78.3%
MgF_2_ [74]	coating	PE separator	1 M LiPF_6_ in EC/DEC/DMC (1:1:1 *v*/*v*/*v*)	NCM811	0.82 mg cm^−2^	2 C 400 cycles 84.5%
Graphene [75]	vacuum filtration	Celgard 2500 separator	1 M LiTFSI in DOL/DME (1:1 *v*/*v*) with 2% LiNO_3_ additive (Li||Cu/Li cell) 1 M LiPF_6_ in EC/DEC/DMC (1:1:1 *v*/*v*/*v*)	NCM811	30.06 mg cm^−2^	/

## Data Availability

Not applicable.
